# Steroid conversion with CYP106A2 – production of pharmaceutically interesting DHEA metabolites

**DOI:** 10.1186/1475-2859-13-81

**Published:** 2014-06-05

**Authors:** Daniela Schmitz, Josef Zapp, Rita Bernhardt

**Affiliations:** 1 Department of Biochemistry, Saarland University, Campus B2 2, Saarbruecken 66123, Germany; 2 Department of Pharmaceutical Biology, Saarland University, Campus C2 2, Saarbruecken 66123, Germany

**Keywords:** Cytochrome P450, Steroid hydroxylase, *Bacillus megaterium*, Whole-cell conversion, Dehydroepiandrosterone, Microbial, CYP106A2

## Abstract

**Background:**

Steroids are lipophilic compounds with a gonane skeleton and play an important role in higher organisms. Due to different functionalizations - mainly hydroxylations - at the steroid molecule, they vary highly in their mode of action. The pharmaceutical industry is, therefore, interested in hydroxysteroids as therapeutic agents. The insertion of hydroxyl groups into a steroid core, however, is hardly accomplishable by classical chemical means; that is because microbial steroid hydroxylations are investigated and applied since decades. CYP106A2 is a cytochrome P450 monooxygenase from Bacillus megaterium ATCC 13368, which was first described in the late 1970s and which is capable to hydroxylate a variety of 3-oxo-delta4 steroids at position 15beta. CYP106A2 is a soluble protein, easy to express and to purify in high amounts, which makes this enzyme an interesting target for biotechnological purposes.

**Results:**

In this work a focused steroid library was screened in vitro for new CYP106A2 substrates using a reconstituted enzyme assay. Five new substrates were identified, including dehydroepiandrosterone and pregnenolone. NMR-spectroscopy revealed that both steroids are mainly hydroxylated at position 7beta. In order to establish a biotechnological system for the preparative scale production of 7beta-hydroxylated dehydroepiandrosterone, whole-cell conversions with growing and resting cells of B. megaterium ATCC1336 the native host of CYP1062 and also with resting cells of a recombinant B. megaterium MS941 strain overexpressing CYP106A2 have been conducted and conversion rates of 400 muM/h (115 mg/l/h) were obtained. Using the B. megaterium MS941 overexpression strain, the selectivity of the reaction was improved from 0.7 to 0.9 for 7beta-OH-DHEA.

**Conclusions:**

In this work we describe CYP106A2 for the first time as a regio-selective hydroxylase for 3-hydroxy-delta5 steroids. DHEA was shown to be converted to 7beta-OH-DHEA which is a highly interesting human metabolite, supposed to act as neuroprotective, anti-inflammatory and immune-modulatory agent. Optimization of the whole-cell system using different B. megaterium strains lead to a conversion of DHEA with B. megaterium showing high selectivity and conversion rates and displaying a volumetric yield of 103 mg/l/h 7beta-OH-DHEA.

## Background

Cytochrome P450 enzymes (P450s) are heme-containing monooxygenases showing an eponymous absorbance maximum at 450 nm of the reduced CO-complex [1]. P450s compose one of the largest and oldest gene families with representatives in all kingdoms of life [2] were they are involved in the biosynthesis and tailoring of natural products like sterols, terpenoids, polyketides, alkaloids, and others [3]. In humans, P450s are, for instance, involved in the biosynthesis of cholesterol, steroid hormones, vitamin D, eicosanoids and prostaglandins; furthermore, they are key enzymes in the biotransformation of xenobiotics in the liver. Cytochrome P450 enzymes belong to the group of external monooxygenases, catalyzing the insertion of one atom of oxygen into a substrate and the concomitant reduction of the second oxygen atom to water. On the basis of this mechanism P450s are able to perform a variety of reactions including N-oxidations, N-, O-, and S-dealkylations, sulfoxidations, epoxidations, peroxidations, dehalogenations, N-oxide reductions, and even C–C bond cleavage [4,5]. Their broad substrate range and reaction spectrum make cytochrome P450 enzymes interesting candidates for industrial purposes. The ability of P450 to introduce hydroxyl groups into non-activated carbon bonds is unique; this spin-forbidden reaction is only scarcely feasible in a regioselective manner by classical chemical means. Hydroxylations are of special interest to pharmaceutical industry, because the insertion of only one hydroxyl group into a certain substrate can dramatically influence its bioavailability, bioactivity or solubility. One famous example is the P450-dependent formation of pravastatin from compactin. Pravastatin exhibits a considerable increased inhibitory activity against the HMG-CoA reductase, the key enzyme in cholesterol biosynthesis [6]. Thus, the assembly of pravastatin using a bioconversion process developed by Sankyo (present Daiichi-Sankyo) represents a successful industrial application of P450s [7]. Another interesting application of P450s is the production of human metabolites either of endogenous origin, like hydroxysteroids, or hydroxylated xenobiotics such as drugs. However, the use of cytochromes P450 is to date mostly limited to the production of high-value fine chemicals or pharmaceutically interesting human metabolites for analytical purposes. At the moment, the productivity of P450 reactions is often not sufficient for the bulk production of chemicals, due to several limitations like their multi-component character, their low stability under process conditions and the consumption of cost-intensive co-factors (NADPH, NADH) in stoichiometric amounts [7-9]. Furthermore, human cytochrome P450s are membrane bound proteins, which are difficult to express and to purify. To overcome at least some of these drawbacks, whole-cell systems are the method of choice to accomplish P450 reactions [8,10].

Steroids are one of the most interesting classes of P450 substrates for pharmaceutical and biotechnological purposes. These terpenoic compounds with a gonane skeleton play a vital role in the human metabolism. Steroids are responsible for the maintenance of blood-pressure and homeostasis, sex development and stress response. Imbalances in the steroid level can have severe pathological effects [11]. Estrogens, for instance, which are produced in adipose tissue, are involved in the formation of post-menopausal breast cancer [12]. From a biotechnological point of view, bacterial steroid hydroxylases have the advantage, in contrast to their eukaryotic counterparts, that they are soluble proteins, which facilitates their recombinant expression and purification. CYP106A2 from *Bacillus megaterium* ATCC13368 is one of only few cloned bacterial steroid hydroxylases which has been investigated since decades and emerged as a potent biocatalyst for the preparative scale production of hydroxysteroids [13]. Most substrates of CYP106A2 are 3-oxo-Δ^4^ steroids, which are hydroxylated mainly in the 15β-position [14-16]. So far, conversions of 3-hydroxy-Δ^5^, ring-reduced or aromatic steroids with CYP106A2 have not been described. Recent studies conducted with this enzyme showed, however, that it is also capable to hydroxylate di- and triterpenoids with diverse backbones. Bleif et al. identified the diterpene resin acid abietic acid and the tetracyclic triterpene 11-keto-β-boswellic acid as substrates of CYP106A2 [17,18]. Screening of a natural product library further broadened the substrate spectrum of this enzyme, with the identification of the less polar triterpenoids dipterocarpol and betuline as substrates [19]. Interestingly, the aforementioned substrates lack a 3-oxo-Δ^4^ motif and dipterocarpol, betuline, and abietic acid do even not contain any keto-en moiety in their structure, which encouraged us to investigate whether CYP106A2 is yet able to hydroxylate non-3-oxo-Δ^4^ steroids. Screening of a focused library consisting of 11 steroidal compounds, among them 3-hydroxy-Δ^5^, A-ring-reduced, aromatic and D-ring-modified steroids revealed novel non-3-oxo-Δ^4^ steroid substrates for CYP106A2, among them dehydroepiandrosterone (DHEA) and pregnenolone (PREG). NMR analysis showed that both DHEA and PREG were hydroxylated preferably at position 7β. DHEA was also hydroxylated at position 7α, but to a minor extend. 7-oxygenated steroids and sterols are widespread in mammals, birds, fish and plants [20]. In humans, DHEA and PREG are 7α-hydroxylated in various tissues by different cytochrome P450 enzymes including liver, intestine, and brain. However, there is no mammalian enzyme described yet which catalyzes the formation of 7β–OH–DHEA. Experimental and clinical data indicated that hydroxylated DHEA derivatives are bioactive human metabolites, responsible for at least some of the DHEA actions in the body, like neuroprotective, anti-inflammatory and anti-proliferative effects [21,22]. For these reasons we established in this work a competitive P450 whole-cell process for the production of 7β-hydroxylated DHEA.

## Results and discussion

### Screening of a focused steroid library

Most CYP106A2 substrates contain a 3-oxo-Δ^4^-moiety and it was assumed for a long time that this motif is a prerequisite for conversions using this enzyme (Table 1). However, recent findings revealed that CYP106A2 indeed is able to convert compounds lacking such a motif [17,18,23], which encouraged us to investigate whether CYP106A2 is also able to catalyze the hydroxylation of steroids lacking the 3-oxo-Δ^4^-moiety. Therefore, a focused steroid library, consisting of 11 non-3-oxo-Δ^4^ steroids was screened in an *in vitro* enzyme assay. The mitochondrial electron transfer system from bovine adrenals was used to reconstitute the CYP106A2 enzyme system, because the bovine electron transfer proteins are well established as efficient electron suppliers for CYP106A2 and other bacterial P450s [24,25].

**Table 1:** Steroids tested for conversion with CYP106A2

**Steroid**	**Product formation: main hydroxylation position (minor products if known)**	**Induction of high-spin shift**
3-oxo-Δ^4^ type steroids		
Deoxycorticosterone^a^	15β	Yes
6-Fluoric-methyl-deoxycorticosterone^b^	15β	Yes
Corticosterone^a^	15β	No
Cholestenone^c^	15β	Yes
Progesterone^a^	15β (11α, 6β, 9α)	No
17α-Hydroxyprogesterone^a^	15β	No
20α-Dihydroprogesterone^a^	15β	No
Androstenedione^a^	15β	Yes
Testosterone^a^	15β	Yes
3-hydroxy-Δ^5^-steroids		
Dehydroepiandrosterone^†^	7β (7α)	No
Pregnenolone^†^	7β	No
Stigmasterol^†^	No	No
Cholesterol^†^	No	No
Other steroids		
Estradiol^†^	No	No
Estrone^†^	No	No
Cis/Trans-androsterone^†^	No	No
Digitoxigenin^†^	n.d	No
Prednisone^†^	n.d.	No
Dexamethasone^†^	n.d	No

no = no conversion/no spin shift, n.d. = hydroxylation position not determined.

^†^ = tested in this work.

^a^[14]^b^[44]^c^[26].

**Scheme 1:**
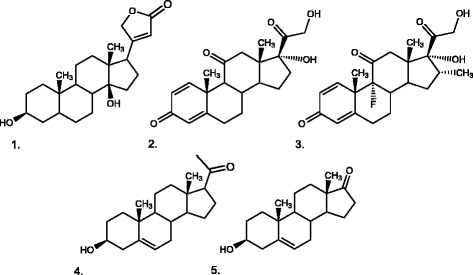
Structures of novel CYP106A2 substrates: digitoxigenin (1), prednisone (2), dexamethasone (3), pregnenolone (4), dehydroepiandrosterone (5) Structures of novel CYP106A2 substrates: digitoxigenin (1), prednisone (2), dexamethasone (3), pregnenolone (4), dehydroepiandrosterone (5).

To investigate the catalytic activity of CYP106A2 towards 3-hydroxy-Δ^5^ steroids, we performed *in vitro* reactions with dehydroepiandrosterone, pregnenolone, cis-androsterone, trans-androsterone, cholesterol, and stigmasterine. Estrone, estradiol, as well as prednisone and dexamethasone were tested as examples for aromatic and ring-reduced steroids. Additionally, we chose digitoxigenin, the steroidal aglycone of the cardio-active glycoside as a potential substrate with D-ring modification. TLC and HPLC analysis were performed and steroid conversion has been observed for five out of eleven substances tested (Table 1). Reaction products were detected using dehydroepiandrosterone (DHEA), pregnenolone (PREG), dexamethasone, prednisone, and digitoxigenin as substrates (Scheme 1). According to these results, it can be stated that the 3-oxo-Δ^4^ moiety is not crucial for steroid conversion with CYP106A2. However, estrone, estradiol, stigmasterol, and cholesterol were not hydroxylated by CYP106A2. We assume that the lower flexibility of the aromatic A-ring of the three steroids hinders binding and hydroxylation. Why cholesterol and stigmasterol are not converted by CYP106A2 cannot be explained at this point. The size of these steroids should not be the problem because CYP106A2 is able to hydroxylate cholestenone and dipterocarpol which are of similar size. Considering that cholestenone is the 3-oxo-Δ^4^ counterpart of cholesterol, it can be assumed that the 3-oxo-Δ^4^ motif present in this case plays a crucial role [26]. Table 1 contains an overview about all steroids which were tested as substrates for CYP106A2.

**Figure 1:**
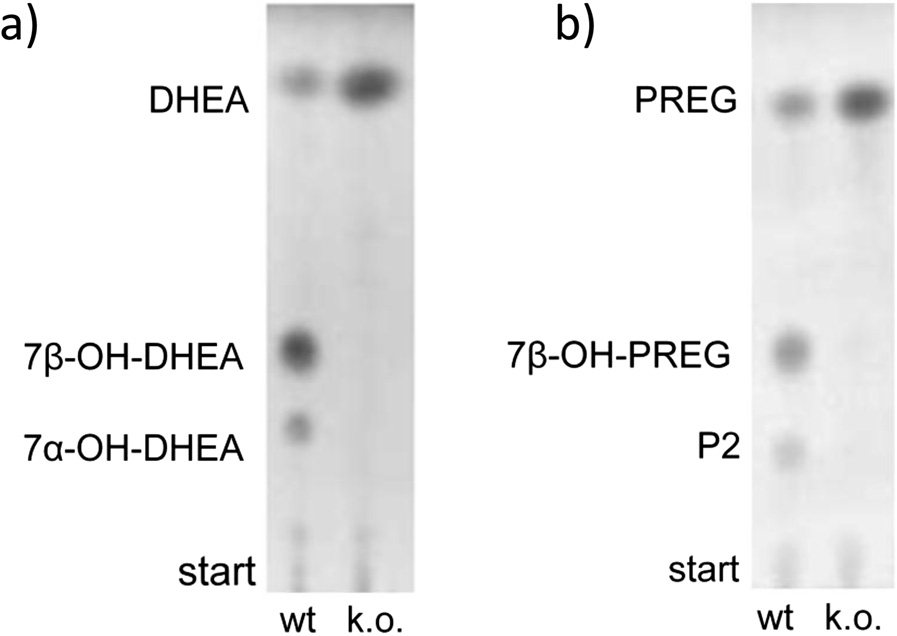
***In vivo *conversion of dehydroepiandrosterone and pregnenolone with *B. megaterium *ATCC13368 and *B. megaterium *ATCC13368 Δ*cyp106a2 *whole cells.** Conversions were performed using 500 μl samples of fresh 16 h cultures incubated with 200 μM of the respective steroid for 60 min at 30°C and vigorous shaking in 1.5 ml reaction tubes. Reaction was quenched and extracted with 500 μl ethyl acetate. DHEA was hydroxylated into 7β-OH-DHEA and 7α-OH-DHEA **(a)**, and PREG was converted into 7β-OH-PREG and an unknown product P2 **(b)**. The *cyp106A2*-knockout strain converted neither DHEA nor PREG **(a,b)**.

NMR spectroscopy was applied to resolve the structure of the newly identified CYP106A2 reaction products. To obtain the required amounts (mg), *B. megaterium* ATCC13368 was used for preparative scale whole-cell conversions. To investigate whether the *B. megaterium* ATCC13368 whole-cells are capable to hydroxylate DHEA, PREG, dexamethasone, prednisone and digitoxin, 500 μl aliquots of fresh over-night cultures were used for small scale conversions. For this purpose, 200 μM of each substrate was added to one 500 μl aliquot and incubated for 60 minutes at 30°C under vigorous shaking. TLC and HPLC analysis revealed that CYP1062-dependent product formation was only detected with PREG and DHEA as substrate (Figure 1). Dexamethasone was not converted in noteworthy amounts by *B. megaterium* ATCC13368 (Additional file 1: Figure S1). Digitoxigenin and prednisone were converted in the whole-cell system, but in their case product formation was also observed using the CYP106A2-deficient knockout strain (Additional file 1: Figures S2 and S3). Because no evident CYP106A2-dependent conversion was detected for dexamethasone, prednisone and digitoxigenin, these substrates were not investigated in more detail in this work. The interference of other *B. megaterium* enzymes or low permeability of the cell membrane for these substrates are possible reasons why their whole-cell conversions were not as promising as those of DHEA and PREG. To circumvent interference with *B. megaterium* enzymes, efficient *E. coli* based whole-cell systems are an alternative in this case [23,27].

**Scheme 2:**
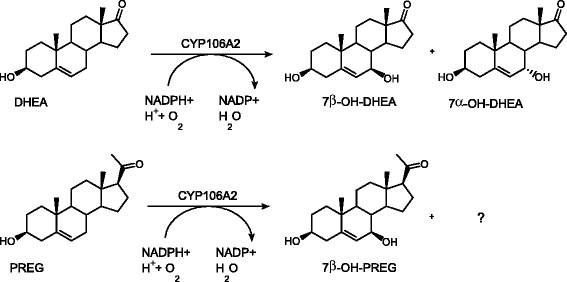
Reaction of dehydroepiandrosterone and pregnenolone with CYP106A2 Reaction of dehydroepiandrosterone and pregnenolone with CYP106A2.

In contrast to the ring-reduced steroids, DHEA and PREG were converted by *B. megaterium* ATCC13368 in sufficient amounts to conduct NMR-analysis. According to the TLC analysis, the DHEA conversion resulted in the formation of two products (Figure 1). The main reaction product (Rf 0.43) was identified with NMR-spectroscopy as 7β–OH-DHEA and the minor reaction product (Rf 0.24) as the 7α stereoisomer (Scheme 2). Whole-cell conversion of PREG with *B. megaterium* ATCC13368 resulted also in the formation of two reaction products (Rf 0.42 and 0.23) (Figure 1). However, in this case only the main product (Rf 0.4) was obtained in sufficient amount and purity for NMR-analysis and was identified as 7β–OH-PREG. Neither DHEA nor PREG reacted with the *cyp106a2*-knockout strain, why we presume the reaction being strictly CYP106A2 dependent in *B. megaterium* whole-cells (Figure 1). Because its conversion was most promising, we choose DHEA for further investigations.

### CYP106A2-dependent *in vitro* hydroxylation of DHEA

**Figure 2:**
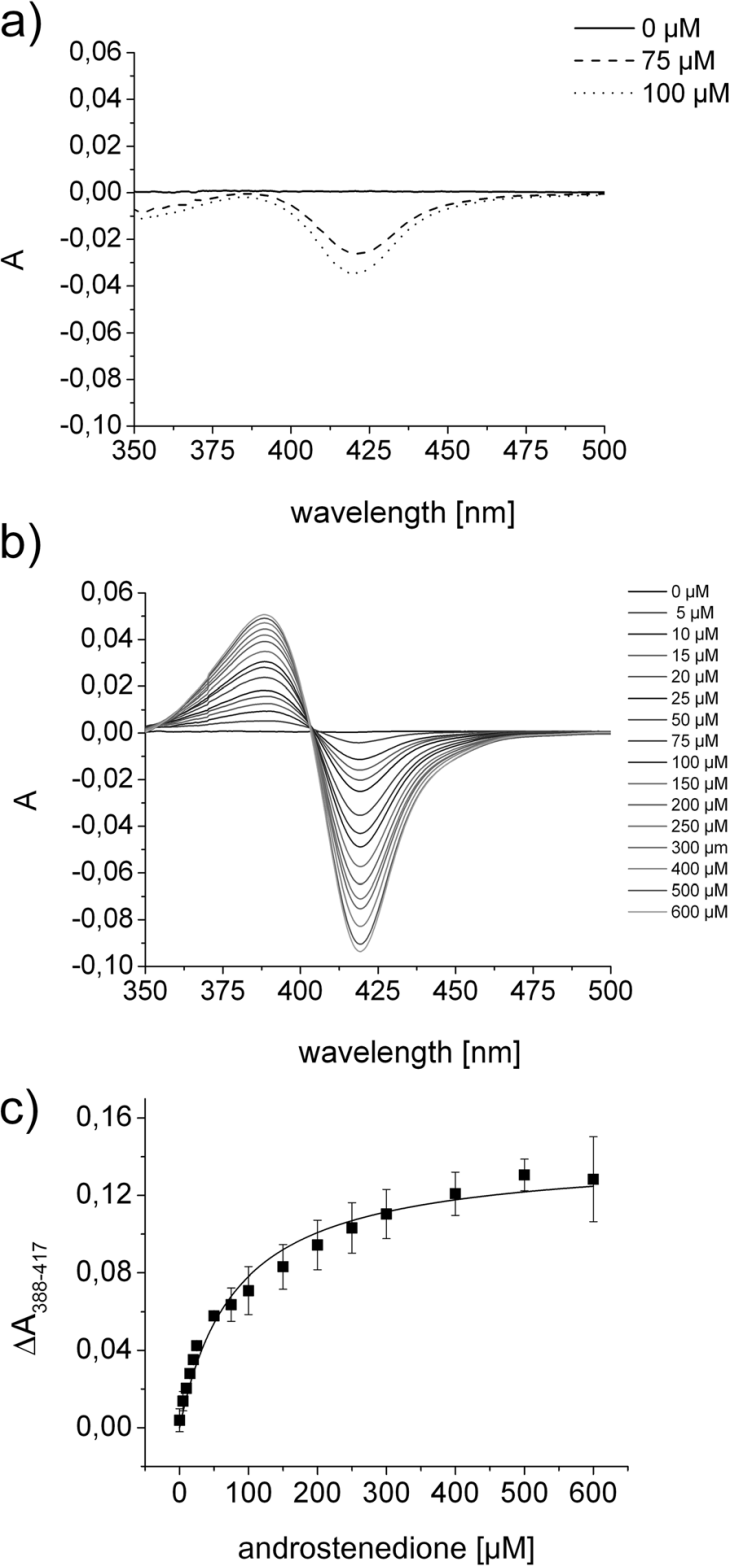
**Difference spectroscopy measurements of DHEA and androstenedione binding to CYP106A2. a)** Difference spectra of DHEA with CYP106A2 in tandem cuvettes recorded from 350 to 500 nm. 0, 75, and 100 μM DHEA solved in DMSO were used. **b)** Difference spectra of androstenedione recorded from 350 to 500 nm in tandem cuvettes. Androstenedione was titrated from 0 to 600 μM into a 10 μM CYP106A2 solution. **c)** The binding constant K_D_ was calculated by plotting the peak-to-through difference and subsequent hyperbolic fit (solid line). The apparent dissociation constant of androstenedione was calculated to be K_D_ 92 ± 11 μM. The results represent mean values and standard deviations from three independent titrations.

The binding of DHEA to CYP106A2 was analyzed *in vitro* using difference spectroscopy. DHEA did not cause any spectral shift in our assay prompting the assumption that the steroid is not able to remove the axial water from the heme during binding (Figure 2a). Interestingly, the 3-oxo-Δ^4^ counterpart of DHEA, androstenedione, induced a high-spin shift in our assay (Figure 2b) and could be used to determine the dissociation constant of androstenedione with CYP106A2 to be 92 ± 11 μM (Figure 2c). It is likely that the keto-group at position 3 in the A-ring leads to an alternate binding mode of androstenedione, resulting in a position maybe closer to the heme compared to that of DHEA, eventually leading to the displacement of the axial water ligand.

In a next step, the apparent Michaelis-Menten parameters describing the CYP106A2 dependent DHEA hydroxylation have been estimated. The minor product, 7α–OH-DHEA, was produced with an apparent reaction velocity V_*max*_^*app*^ of 41 ± 3.5 nmol product min^-1^ * nmol P450^-1^ and an apparent Michaelis constant K_*M*_^*app*^ of 98 ± 22 μM. The reaction leading to the β-hydroxylated product is described by a K_*M*_^*app*^ of 168 μM with an apparent maximal reaction velocity V_*max*_^*app*^ of 71 nmol product min^-1^ * nmol P450^-1^. In comparison, the 15β-hydroxylation of progesterone is approximately five times faster with a V_*max*_^*app*^ of 337 nmol product min^-1^ nmol P450^-1^. The conversions of 11-deoxycorticosterone and 11-deoxycortisol are within the same order of magnitude as the 15β-hydroxylation of progesterone with a V_*max*_^*app*^ of 246 nmol product min^-1^ nmol P450^-1^[28], and 172 nmol product min^-1^ nmol P450^-1^[29]. Bleif et al. showed that the conversion of triterpene acids like the abietic acid and the 11-keto-β-boswellic acid is slower than that of the 3-oxo-Δ^4^-steroids, with V_*max*_^*app*^ values of 22 and 97 nmol product min^-1^ nmol P450^-1^, respectively [17,18]. Thus, the velocity of the CYP106A2-dependent hydroxylation of DHEA is comparable to the hydroxylation of terpene acids with this enzyme.

### CYP106A2-dependent *in vivo* hydroxylation of DHEA

#### Evaluation of DHEA whole-cell conversion

**Figure 3:**
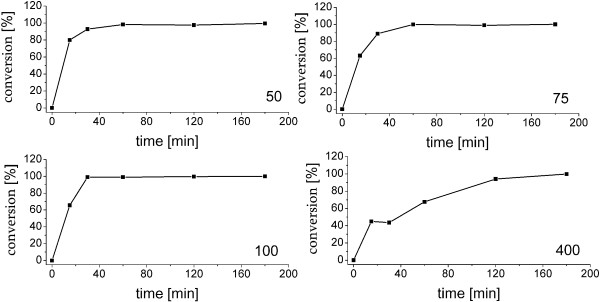
**Time- and concentration- dependent DHEA conversions using *B. megaterium *ATCC13368 whole-cell bioconversion.** 50 ml *B. megaterium* WT cultures were incubated at 30°C and 150 rpm for 16 hours prior to substrate addition. 50, 75, 100 and 400 μM DHEA (max. 5% ethanol v/v) were added into the cultures and reactions were followed using TLC analysis. Samples were taken at indicated time points, (500 μl) quenched, and extracted with 500 μl ethyl acetate. The organic phase was separated, evaporated to dryness, and chromatographed using ethyl acetate as mobile phase on TLC-plates. After anisaldehyde staining the relative substrate consumption in % was calculated using the spot intensity with the Image-Lab™ (BIO-RAD, Germany) software.

**Figure 4:**
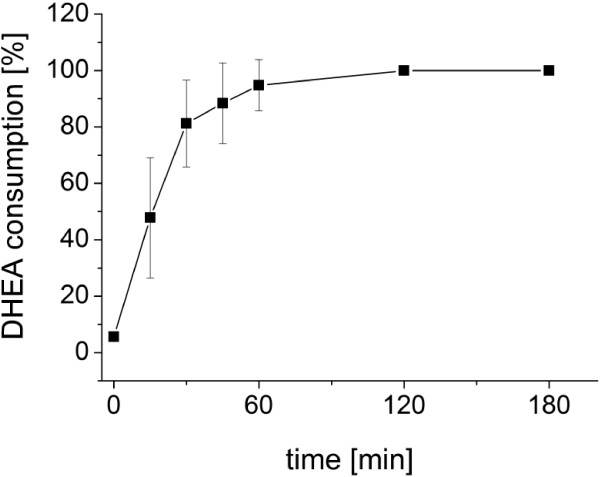
**Conversion of 400 μM DHEA.** 50 ml *B. megaterium* ATCC13368 cultures were incubated 16 hours at 30°C and 150 rpm. DHEA (400 μM dissolved in ethanol) was added and cultures were incubated additional 180 min. 500 μl samples were taken at 0, 15, 30, 45, 60, 120 and 180 minutes after substrate addition. Samples were quenched and extracted with 500 μl ethyl acetate, evaporated to dryness and chromatographed using ethyl acetate as mobile phase. After anisaldehyde staining the relative substrate consumption in % was calculated using the spot intensity with the Image-Lab™ (BIO-RAD, Germany) software. The graph shows the mean values and standard deviation from three independent conversions.

As mentioned above, cytochrome P450 *in vitro* reactions are not suitable for preparative scale production of hydroxysteroids [8]. Therefore, our aim was to establish an effective whole-cell system for the efficient production of 7β-OH-DHEA. We used *B. megaterium* ATCC13368, which expresses CYP106A2 in the stationary phase of growth and has been successfully used for preparative-scale production of CYP106A2 reaction products in the past, with higher susceptibility for terpenoic substrates than *E.coli*[18,19]. As described above, the *B. megaterium* strain ATCC13368 approved to be suitable for *in vivo* mg scale 7β-hydroxylation of DHEA. To evaluate the efficiency of DHEA hydroxylation, time-dependent and substrate concentration-dependent bioconversions were performed. Steroids are lipophilic compounds and hence scarcely soluble in water. In order to increase the solubility of DHEA, ethanol was used as co-solvent. However, ethanol is toxic to bacterial cells why its use as solvent is limited in whole-cell biotransformations. It was found that the growth and viability of *B. megaterium* was not influenced by ethanol concentrations up to 5% (data not shown). Using 5% ethanol as co-solvent, the solubility of DHEA was increased to 400 μM. Finally, DHEA concentrations ranging from 50 to 400 μM DHEA were added to 50 ml cultures and the reaction was followed until the substrate was consumed (Figure 3). Complete substrate consumption was observed for all tested concentrations. 50, 75 and 100 μM DHEA were consumed within one hour (Figure 3). The highest concentration tested, 400 μM, was completely consumed within three hours. Using 400 μM, fluctuations in the DHEA conversion between the first three measurement points occurred, which are most probably caused by incomplete mixing of the substrate in the culture and an unbalanced uptake of DHEA into the cells. In order to affirm our finding that 400 μM DHEA are consumed completely within 180 minutes the conversion using this concentration was repeated additional three times, in all cases no substrate was detected anymore after 180 minutes, and more than 99% of the substrate was consumed within 120 minutes (Figure 4.).

#### Discontinuous DHEA-feed

**Figure 5:**
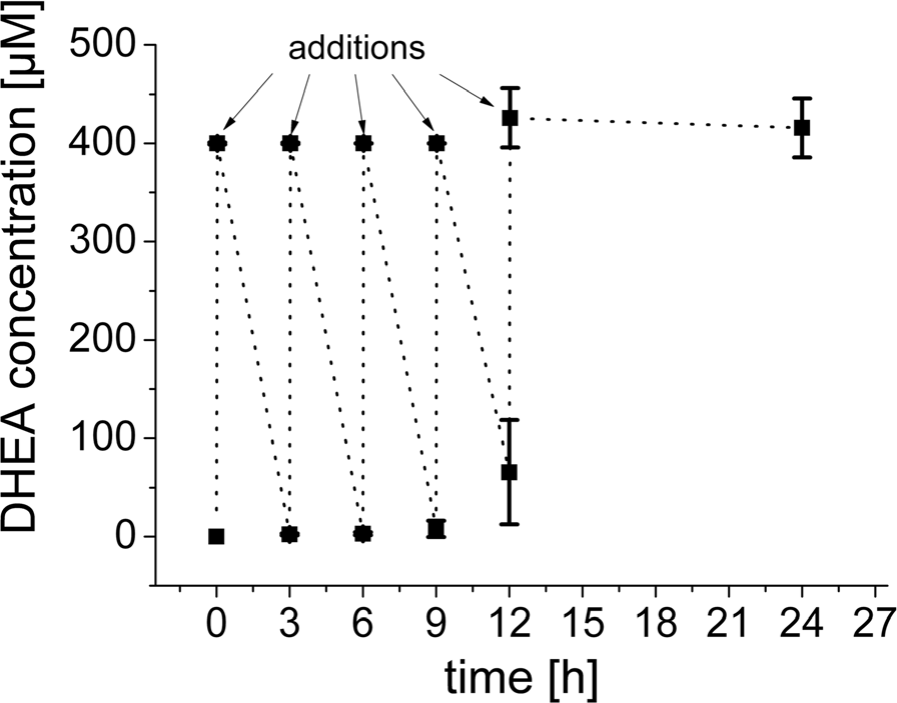
**Discontinuous feed of DHEA to *B. megaterium *ATCC13368.***B. megaterium* ATCC13368 cultures were incubated at 30°C and 150 rpm in broth. After 16 h cultivation 400 μM DHEA was added (0 h) 5 times with 3 hours break between the individual additions. After the last addition (reaching 2000 μM DHEA in total) the cultures were incubated for additional 12 h. DHEA concentration in the cultures was calculated using TLC analysis and the software ImageLab™ (BIO-RAD, Germany).

Using ethanol as co-solvent we were able to dissolve 400 μM DHEA in *B. megaterium* ATCC13368 cultures. Using higher DHEA concentrations than 400 μM, however, aggregation of the substrate was observed. Whereas, solid substrate does not necessarily circumvent product formation, substrate aggregation lowers the conversion rate. To avoid substrate aggregation in the media, a discontinuous feed of DHEA into the reaction was investigated. Because 400 μM DHEA are completely converted by *B. megaterium* ATCC13368 within 180 minutes (Figures 3 and 4), DHEA was fed in 400 μM portions to the cells with three hours break between the individual additions. DHEA concentrations in the cultures were measured before and immediately after the substrate additions. The first two feeds after 3 and 6 hours resulted in complete DHEA consumption, meaning a total conversion of 800 μM DHEA within 6 hours. After the third feeding step, complete conversion was detected in two of three flasks, resulting in a mean conversion of 98 ± 2%. The conversion rate dropped further after the addition, within the next 3 hours only 82 ± 14% DHEA were hydroxylated. The last 400 μM DHEA were not converted into hydroxylated products within the following 12 hours (Figure 5). Altogether, *B. megaterium* ATCC13368 was able to hydroxylate approximately 1.5 mM DHEA within 12 hours, resulting in a conversion rate of 438.4 mg*l^-1^ (Figure 5). Although substrate accumulation was avoided, we encountered limitations of the *B. megaterium* ATCC13368 bioconversion. It can be assumed that the decrease in activity is caused either by direct inhibition of CYP106A2 or indirectly through negative effects of the accumulating products on the *B. megaterium* ATCC13368 metabolism.

To answer this question, the hydroxylation activity of *B. megaterium* ATCC13368 cultures was assessed over a time-period of 48 hours. For this purpose, cells were cultivated in broth at 30°C and 500 μl aliquots were sampled after 16, 24, 36, and 48 hours. Conversions were performed with 100 μM substrate using 500 μl aliquots in 1.5 ml reaction tubes at 30°C for 30 minutes. The activity of the cells which were incubated 16 hours prior to the activity assay was defined as 100% and the activity of the aliquots taken after 24, 36 and 48 hours was calculated relative to this value. No decrease in the hydroxylation efficiency of *B. megaterium* ATCC13368 was observed within 36 hours (Table 2). On the contrary, the mean conversion, relative to the activity measured after 16 hours, increased to 136%, although it has to be taken into account that the standard deviation was quite high. Nevertheless, it can be concluded, that the hydroxylation efficiency of *B. megaterium* ATCC13368 is constant within 36 hours and did not decrease in our experiments until 48 hours. We therefore assume that the limitation observed in the discontinuous feeding reaction is caused by the accumulation of hydroxysteroid in the medium. However, with 438.4 mg*l^-1^ DHEA converted within 12 hours (e.g. 36 mg*l^-1^*h^-1^) the volumetric productivity of the systems achieves the minimum process requirements for the industrial production of pharmaceuticals, which is estimated to 1 mg*l^-1^*h^-1^[30].

**Table 2:** **Hydroxylation activity of *B. megaterium *** **ATCC13368 monitored over a period of 48 hours**

**16 h**	**24 h**	**36 h**	**48 h**
100%	105 ± 3%	136 ± 42%	84 ± 22%

Cells were cultivated in 50 ml Erlenmeyer flasks at 30°C and 150 rpm. 500 μl samples were taken at time points indicated. Conversions were performed with 100 μM substrate in 1.5 ml reaction tubes at 30°C for 60 min. The reactions were quenched and extracted with ethyl acetate and the steroid conversion was analyzed with HPLC. The activity of *B. megaterium* after 16 h cultivation was defined as 100%. Results are shown as mean values and deviations are from two independent experiments.

#### Whole-cell conversion using resting cells of *Bacillus megaterium*

**Figure 6:**
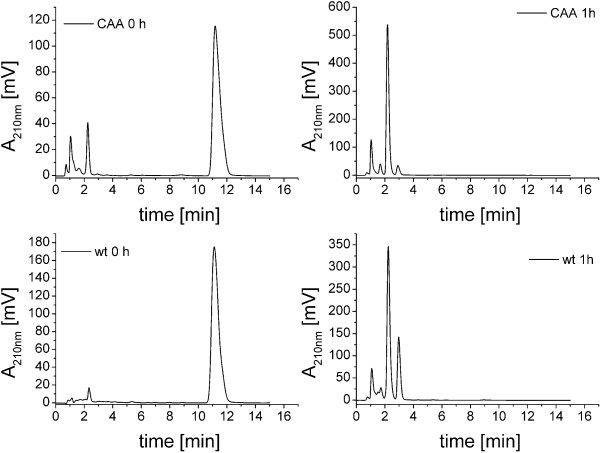
**HPLC chromatograms of DHEA conversion using resting cells *B. megaterium *ATCC13368 (wt) and MS941_pSMF2.1CAA (CAA).** Cultures were incubated at 30°C and 150 rpm for 24 h, washed once and suspended in 50 ml potassium phosphate buffer adjusted to pH 7.4. Heterologous protein expression in *B. megaterium* MS941_pSMF2.1CAA cells was induced with 0.5% (w/v) xylose. DHEA (400 mM) was added as ethanolic solution (5% v/v). Samples were taken directly after substrate addition (CAA 0 h, wt 0 h) and after 1 h (CAA 1 h, wt 1 h). No DHEA (R_T_ = 11.5 min) was detected in the 1 h samples.

Although an efficient conversion rate was achieved with 438 mg l^-1^ by stepwise addition of DHEA to the cultures, the use of whole cells in complex medium hampered the analysis and subsequent chromatography of the reaction products, because of huge amounts of media constituents and *B. megaterium* metabolites. Resting cells were used to overcome this problem. An additional advantage of resting cells is the avoidance of metabolites which accumulate during bacterial growth and might inhibit the enzyme reaction. *E. coli* for instance produces indole by metabolizing tryptophan as carbon source if sugar is depleted and this indole in turn can act as cytochrome P450 inhibitor in whole-cell bioconversions [27,31]. However, resting cells are not always better suited for bioconversions than growing cells. For the bioconversion of DHEA with *B. megaterium* resting cells, 50 ml cultures were incubated 16 hours at 30°C and 150 rpm in complex medium. Afterwards, cells were centrifuged, washed once and suspended in 50 ml potassium phosphate buffer (50 mM pH adjusted to 7.4). 400 μM DHEA were added to the reaction mixture and the conversion was followed using rpHPLC analysis. As shown in Figure 6, using resting cells, 400 μM DHEA were completely converted within 60 minutes, whereas it took 180 minutes to completely convert the same amount using growing cells (Figures 3 and 4). Taken together, our experiments demonstrate that the conversion rate was considerably increased using *B. megaterium* resting cells compared with growing cells.

#### Conversion of DHEA using the recombinant *B. megaterium* MS941_pSMF2.1CAA strain

**Figure 7:**
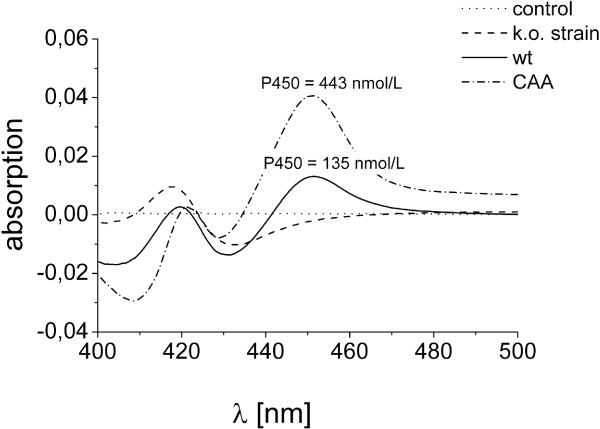
**P450 content in the used *B. megaterium *strains.** CO-difference spectra recorded after 24 hours cultivation of *B. megaterium* ATCC13368 (wt), *cyp106a2*-knockout strain (k.o. strain), and MS941_pSMF2.1CAA (CAA) in crude lysates. Cells were incubated at 30°C and 150 rpm in broth, which was supplemented with 35 μg/ml tetracycline for the cultivation of *B. megaterium* ATCC13368 *cyp106a2*-knockout and *B. megaterium* MS941 cells. The protein expression in MS941_pSMF2.1CAA cells was induced by adding 0.25% xylose to the culture at OD_600_ of 0.4 and subsequent incubation for 24 hours.

*B. megaterium* ATCC13368 expresses CYP106A2 in the stationary phase of growth. The maximum P450 content in our experiments was reached after 24 hours with 135 nmol*l^-1^ culture (Figure 6). No P450 was detected in the respective knock-out strain (Figure 6). For biotechnological purposes, it would be of interest to increase the expression rate of CYP106A2 and its redox partners. However, no inducer of CYP106A2 expression in *B. megaterium* is known so far. In our experiments, DHEA was also not able to induce CYP106A2 expression in *B. megaterium*. In order to increase the CYP106A2 amount in *B. megaterium,* an expression system for CYP106A2 together with its heterologous redox partners AdR and Adx_4-108_ in the strain *B. megaterium* MS941 has been developed in our laboratory [18]. Using this system, the expression rate of P450 averages 450 nmol*l^-1^ culture, displaying a three-fold increase compared to the wild type strain (Figure 7). Although *B. megaterium* MS941 contains four P450s in its genome [32], Western Blot analysis conducted by Bleif et al. showed that the P450 content in the expression system is mainly composed of CYP106A2. Using this overexpression strain, the conversion rate of 11-keto-β-boswellic acid was increased by the factor of four compared with the wild type [18]. Therefore, we aimed to improve the DHEA conversion using the recombinant expression strain *B. megaterium* MS941 overexpressing CYP106A2. According to the results achieved with the ATCC13368 strain (shown in Figure 6), resting cells were used for the bioconversion. Again, 400 μM DHEA was added and samples were taken after 0, 1, 4, 8 and 24 hours. To corroborate the CYP106A2 dependence of DHEA conversion, the MS941 strain without the expression vector was used as control. The control strain did not convert DHEA within 24 h of incubation (Additional file 1: Figure S4). To our surprise, the conversion rate of *B. megaterium* MS941_pSMF2.1CAA cells was comparable to that of resting cells of the ATCC13368 strain, 400 μM DHEA were completely converted after 60 minutes (Figure 6). Obviously, there are other rate-limiting parameters in CYP106A2-dependent DHEA conversion in *B. megaterium,* most probably substrate uptake and product exit, which are known bottlenecks of whole-cell conversions. Interestingly, the product pattern differed in the reactions performed with the ATCC13368 strain from that using the recombinant expression system. The latter shifted the selectivity in favor of the reaction leading to the β-hydroxylated product. The ATCC13368 strain converted DHEA into 70% 7β-OH-DHEA and 30% 7α-OH-DHEA, whereas the MS941_pSMF2.1CAA strain produced 90% 7β-OH-DHEA (Figure 6, Additional file 1: Figure S4). Although no increase in the conversion rate could be measured with the overexpression strain, due to the increased stereoselectivity a time-space yield 103 mg*l^-1^*h^-1^ 7β-OH-DHEA was calculated using this system, reflecting the fastest CYP106A2-dependent hydroxysteroid production described using *B. megaterium* whole-cells up to now.

At this point it cannot be fully explained why the use of the recombinant expression system alters the selectivity of the reaction. However, similar observations have been made with the *E. coli* system for the production of hydroxylated progesterone derivatives using CYP106A2 mutants, were the *in vitro* selectivity differed from that observed in the *in vivo* conversions [33].

## Conclusions

Summarizing the results, we broadened the substrate spectrum of CYP106A2 and identified novel reaction products. Screening of a focused steroid library revealed, for the first time, that CYP106A2 converts 3-hydroxy-Δ^5^ and A-ring reduced steroids. The major group of CYP106A2 substrates still consists of 3-oxo-Δ^4^ steroids, but the present work describes for the first time its ability to hydroxylate pregnenolone (PREG) and dehydroepiandrosterone (DHEA), both mainly at position 7β. Assuming that both ends of the steroid molecule interact with amino acid side chains to position the steroid above the heme, four principal binding modes have been described in the literature; normal, reverse, normal inverted and reverse inverted [34]. The differences in the hydroxylation position of 3-oxo or 3-hydroxy steroids most probably result from alternative binding modes in the active side of CYP106A2, assessing, for instance, the differences between DHEA and androstenedione hydroxylation: The change of the hydrogen-bond-acceptor (=O) into a donor (-OH), at position C-3, may cause an inverted or reversed binding of the molecule. Another possible explanation is that the loss of one hydrogen-bond acceptor loosens the binding at C-3, so that the steroid slips perhaps deeper into the reactive pocked resulting in that the C-15 is moved from and the C-7 towards the heme-iron.

Even though the mechanistic insights of CYP106A2 steroid hydroxylation remain elusive, its production of human DHEA metabolites is highly interesting, because 7-oxygenated DHEA derivatives have been discussed as neuroprotective, immune-modulatory and anti-inflammatory agents [35]. Besides, hydroxylated steroids are highly interesting lead compounds for drug development targeting P450 enzymes, especially CYP19A1 (aromatase) which is involved in the formation of post-menopausal breast cancer. Li et al., for instance, showed that 7-substituted 1,4,6-androstatriene-3,17-diones can act as enzyme-activated irreversible aromatase inhibitors [36]. The bioconversion rates of the systems described achieve appreciable rates, up to 400 μM DHEA within one hour, thus comparable to described fungal systems [37]. To our opinion the major challenge for further optimizations of DHEA bioconversion with *B. megaterium* is the limitation most probably caused by accumulation of hydroxysteroids in the reaction mixture. This can be met by performing the conversions in liquid-liquid biphasic-systems, in which a water-immiscible liquid phase is used as substrate reservoir and product recipient. Such systems have already been successfully conducted with recombinant *E. coli* and solvent tolerant *Pseudomonas putida* strains [38,39]. Yet, combined with the recombinant expression strain, which produced the 7β-stereoisomer with 90% de resulting in a space-time yield of 103 mg*l^-1^*h^-1^ 7β-OH-DHEA, the present systems provides an excellent starting point to establish biotechnological processes for the production of high-value hydroxysteroids.

## Material and methods

### Chemicals and solvents

All chemicals used in this work were from standard sources and of highest purity available. Solvents used for chromatography were of gradient grade, solvents used for large scale extraction were of analytical purity. The focused steroid library used in this work consisted of: dehydroepiandrosterone (DHEA), pregnenolone (PREG), estradiol, estrone, dexamethasone, prednisone, androstenedione, stigmasterol, cholesterol and cis-, trans-androsterone. All steroids were obtained from Sigma-Aldrich (Sigma-Aldrich Biochemie GmbH, Germany) and were of highest purity available.

### *Bacillus megaterium*: strains plasmids and cultivation

*Bacillus megaterium* ATCC13368, the host strain of CYP106A2, as well as a respective knockout strain *Bacillus megaterium* ATCC13368 Δ*cyp106A2*, were both kind gifts of Dr. R. Rauschenbach (Schering AG, Berlin, Germany). *Bacillus megaterium* MS941 was kindly provided by Prof. Dieter Jahn (Institute of Microbiology, TU Braunschweig, Germany). Besides, we used a xylose-inducible recombinant expression system, which has recently been established in our group, to co-express CYP106A2 and its heterologous redox-partners adrenoxodin (Adx) and adrenodoxin reductase (AdR) using the expression vector pSMF2.1_CAA in *B. megaterium* MS941 [18].

Unless otherwise noted, *Bacillus megaterium* cells were cultivated in complex medium consisting of 25 g yeast extract (Becton, Dickinson and Company, USA), 12 g soytone (Becton, Dickinson and Company, USA) and 5 ml glycerol buffered with potassium phosphate buffer (2.31 g KH_2_PO_4_, 12.5 g K_2_HPO_4_) adjusted to pH 7.4 in 1 l distilled water. Cultivations were generally conducted at 30°C and 150 rpm in baffled shake-flasks (300 ml or 2 l). For the cultivation of MS941 cells transformed with the plasmid pSMF2.1_CAA and for the cultivation of the ATCC13368 Δ*cyp*106*a*2-strain the medium was supplemented with 10 mg*l^-1^ tetracycline.

### Protein expression and purification

CYP106A2, a truncated form of bovine adrenodoxin (Adx_4-108_) and bovine adrenodoxin reductase (AdR) were expressed and purified as described elsewhere [19,40,41].

### *In vitro* conversion and catalytic activity

Substrate conversions with CYP106A2 were conducted using an *in vitro* reconstituted system with bovine Adx_4-108_ (5 μM) and bovine AdR (0.5 μM) as redox partners for CYP106A2 (0.25 μM). An NADPH regeneration system consisting of glucose-6-phosphate-dehydrogenase (1 U), glucose-6-phosphate (5 mM) and MgCl_2_ (1 mM) was used for the continuous supply with electrons. Reactions were carried out in 1.5 ml reaction tubes at 30°C and 750 rpm in 50 mM HEPES-buffer adjusted to pH 7.4 and supplemented with 0.05% (v/v) Tween 20. The total reaction volume was 250 μl. Reactions were started by adding 500 μM pre-warmed (30°C) NADPH dissolved in 50 mM HEPES-buffer. For the endpoint-kinetics the reactions were incubated for two minutes. The reaction times were ten minutes in order to screen the steroid library for novel CYP106A2 substrates. Reactions were stopped and extracted twice with 500 μl ethyl acetate. The organic phases were pooled, evaporated to dryness and subsequently chromatographed via reversed-phase high-performance-liquid-chromatography (rpHPLC).

### Spectroscopic binding assay

Difference spectroscopy was used to investigate the binding behavior of DHEA and androstenedione to CYP106A2. For this purpose, tandem cuvettes were used as described in the literature [42,43]. In one chamber of each cuvette 10 μM enzyme in 50 mM potassium phosphate buffer (pH 7.4) was added, whereas buffer without enzyme was filled into the other chamber. After performing the baseline correction, steroids were titrated into the enzyme solution of the measurement cuvette and into the chamber containing only buffer of the reference cuvette. The solvent DMSO alone was titrated vice versa to avoid falsification of the measurement according to imbalanced dilution of the enzyme solution. After baseline correction, the difference spectra were recorded from 300 to 500 nm. For the steroids 5 mM, 10 mM and 40 mM stock solutions were prepared using DMSO as solvent. Titrations were performed using a maximal concentration of 2% DMSO (v/v) and final steroid concentrations ranging from 0 – 600 μM. The K_D_ value was calculated by plotting the peak-to-through differences ΔA (380 nm – 417 nm) against the total ligand concentration. The apparent equilibrium dissociation constant was calculated by hyperbolic regression of the resulting plot (y = (P_1_x/P_2_ + x) with P_2_ = K_D_) using the origin software version 8.6 (OriginLab Corporation, Massachusetts, USA).

### Whole-cell biotransformations

Whole cell bioconversions were performed with *B. megaterium* ATCC 13368 and *B. megaterium* MS941_pSMF2.1CAA cultures. As control a Δ*cyp106a2* knock-out strain *of B. megaterium* ATCC 13368 [44] and the MS941 strain without the expression plasmid for CYP106A2 were used. In each case pre-cultures were prepared by inoculating 50 ml broth (25 g yeast extract, 12 g soytone, 5 ml glycerine and no further additives) with 50 μl of deep-frozen stocks and incubated 24 hours at 30°C and 150 rpm in baffled flasks. Main cultures were inoculated by diluting an aliquot of the pre-cultures 1 to 100 with fresh medium. For the incubation of *B. megaterium* ATCC 13368 cells the cultures were incubated 16 h depicting the stationary phase of growth, to ensure the expression of CYP106A2. To express CYP106A2 with *B. megaterium* MS941 strain transformed with the expression plasmid pSMF2.1_CAA, 0.25% (w/v) xylose were added for induction when the main culture reached OD_600_ of 0.4. Protein expression was conducted for 24 hours after induction as described [18]. The 16 h cultures of the ATCC13368 and the 24 h cultures of the MS941_pSMF2.1CAA strain, consistent with an OD of 20 and approximately 40 g-wcw per liter were used for whole-cell conversions. Substrates were generally added as ethanolic solutions, with the final ethanol concentration not exceeding 5% (v/v). Unless otherwise stated, conversions were performed with 50 ml cultures in 300 ml baffled shake flasks at 30°C and 150 rpm using the soytone containing broth or 50 mM potassium phosphate buffer (resting cell conversions). Samples were taken at indicated time points; reactions were quenched and concomitantly extracted with 500 μl ethyl acetate. To follow the CYP106A2 catalyzed reactions, the organic phases were collected, evaporated to dryness, and analyzed via HPLC or TLC.

For the preparative scale conversions of dehydroepiandrosterone (DHEA) and pregnenolone (PREG), 1 L cultures, distributed to five 2 L baffled shake flasks (200 ml each), were inoculated with 1 ml of a deep-frozen stock (200 μl each) and cultivated for 16 h at 30°C and 150 rpm. 115 mg of DHEA and 120 mg of PREG, respectively, were dissolved in 50 ml ethanol and added to the cultures after 16 h incubation. Samples were taken every two hours and reactions were monitored using TLC analysis. When the substrate was completely consumed, the cultures were pooled and extracted three times with 500 ml ethyl acetate. The organic phases were combined, dried and the resulting residue was chromatographed via a silica gel (60) column, using ethyl acetate as mobile phase. 10 ml fractions were collected and analyzed via TLC; homogenous fractions containing the reaction products were pooled and evaporated to dryness. Because of a yellowish impurity, the main product of the DHEA reaction was additionally chromatographed via a silica gel column using a mixture of hexane/ethyl acetate/methanol in a ratio of 1:4:0.5 as mobile phase. The resulting homogeneous fractions were evaporated to dryness and analyzed by TLC. Those products which were obtained in sufficient amounts and purity were analyzed by NMR spectroscopy. The yields after silica gel chromatography were 46 mg for the first DHEA reaction product (chromatographed twice), 23 mg for the second reaction product and 42 mg for the main pregnenolone product and approximately 1 mg for the second pregnenolone product.

### Chromatographic methods

TLC was used to monitor the *in vivo* biotransformation using growing cells in complex medium and for the analysis of homogenous fractions obtained after silica gel column chromatography. Samples were spotted onto TLC aluminum plates (4 x 8 cm; silica 60; layer thickness, 0.2 mm; Roth, Karlsruhe, Germany), and developed once in a rectangular solvent tank containing ethyl acetate as mobile phase. Spots were detected using an anisaldehyde dipping bath (4-methoxybenzaldehyde, 5 ml; sulfuric acid, 2.5 ml; glacial acetic acid, 3.75 ml; ethanol, 278.75 ml), the reaction of anisaldehyde-sulfur reagent with the steroids and terpenoids to colored products was started by heating the plates to 100°C using a hot air gun.

Reversed-phase high-performance-liquid-chromatography (HPLC) was performed using a Jasco system (Pu-980 HPLC pump, an AS-950 sampler, and UV-975 UV/visible detector, and a LG-980-02 gradient unit from Jasco, Gross-Umstadt, Germany). A reversed phase ec MN Nucleodor C18 (5 μm, 4.0 × 125 mm) column (Macherey-Nagel, Bethlehem, PA, USA) was used. A mixture of acetonitrile and water (40:60) served as mobile phase. Steroids containing a keto-en moiety were detected at 254 nm. From the set of steroids without keto-en-moiety only DHEA was monitored with rpHPLC at 210 nm, but only after conversion *in vitro* or with resting cells, because the samples from whole-cell conversions in complex-media included to many impurities, hampering the rpHPLC analysis at 210 nm. In all measurements the column oven temperature was kept at 40°C and the flow rate of the mobile phase was 1 ml*min^-1^.

### NMR spectroscopy

NMR spectra were recorded in CDCl_3_ at 300 K with a Bruker DRX 500 NMR spectrometer. The chemical shifts were given relative to CDCl_3_ at *δ* 77.00 (^13^C NMR) or CHCl_3_ at *δ* 7.24 (^1^H NMR) using the standard *δ* notation in parts per million (ppm). The 2D NMR spectra were recorded as gs-HHCOSY, gs-NOESY, gs-HSQCED and gs-HMBC.

The conversion of DHEA led to the production of the known 7*α*-OH- and 7*β*-OH-DHEA epimers. Their structures followed directly from the comparison of their NMR data with those from literature [45].

#### 3β,7α-Dihydroxyandrost-5-en-17-one (7α-OH-DHEA)

^1^H NMR (CDCl_3_, 500 MHz) : *δ* 0.86 *s* (3H, 3x H-18), 0.99 *s* (3H, 3x H-19), 1.09 *m* (H-1a), 1.25 *m* (H-12a), 1.26 *m* (H-9), 1.48 *m* (H-11a), 1.49 *m* (H-2a), 1.55 *m* (H-15a), 1.64 *m* (H-8), 1.68 *m* (H-11b), 1.77 *m* (H-14), 1.80 m (H-12b), 1.83 *m* (H-2b), 1.85 *m* (H-1b) 2.08 m (H-15b), 2.12 *m* (H-16a), 2.27 m (H-4a), 2.34 m (H-4b), 2.45 m (H-16b), 3.54 m (H-3), 3.94 *dd* (*J* = 5.0 and 5.0 Hz, H-7), 5.61 *dd* (5.0 and 2.0 Hz, H-6). ^13^C NMR (CDCl_3_, 125 MHz): *δ* 13.29 (C-18), 18.28 (C-19), 20.09 (C-11), 21.93 (C-15), 31.07 (C-12), 31.30 (C-2), 35.80 (C-16), 36.97 (C-1), 37.22 (C-10), 37.53 (C-8), 41.95 (C-4), 42.62 (C-9), 44.96 (C-14), 47.11 (C-13), 64.28 (C-7), 71.17 (C-3), 123.57 (C-6), 146.57 (C-5), 221.10 (C-17).

#### 3β,7β-Dihydroxyandrost-5-en-17-one (7β-OH-DHEA)

^*1*^*H* NMR (CDCl_3_, 500 MHz): *δ* 0.88 *s* (3H, 3x H-18), 1.05 *m* (H-1a), 1.06 *s* (3H, 3x-H19), 1.08 *m* (H-9), 1.23 *m* (H-12a), 1.42 m (H-14), 1.48 *m* (H-11a), 1.50 *m* (H-2a), 1.55 *m* (H-8), 1.68 *m* (H-11b), 1.82 *m* (H-12b), 1.83 *m* (H-15a), 1.84 *m* (H-1b), 1.85 *m* (H-2b), 2.09 *m* (H-16a), 2.22 *m* (H-15b), 2.24 *m* (H-4a), 2.33 *ddd* (J = 13.5, 5.0 and 2.0 Hz, H-4b), 2.45 *m* (H-16b), 3.53 *m* (H-3), 3.93 *ddd* (*J* = 8.0, 2.0 and 2.0 Hz, H-7), 5.29 *dd* (*J* =2.0 and 2.0 Hz, H-6). ^13^C NMR (CDCl3, 125 MHz): *δ* 13.60 (C-18), 19.19 (C-19), 24.21 (C-15), 20.41 (C-11), 31.51 (C-12), 31.51 (C-2), 35.98 (C-16), 36.69 (C-10), 36.90 (C-1), 40.52 (C-8), 41.67 (C-4), 47.78 (C-13), 48.27 (C-9), 51.23 (C-14), 71.27(C-3), 72.88 (C-7), 125.50 (C-6), 143.75 (C-5), 221.11 (C-17).

In contrast to pregnenolone the NMR spectra of its conversion product gave hint to an additional hydroxyl group (*δ* 73.14 (CH) and *δ* 3.83). Its position at C-7 was deduced from HMBC correlations between H-7 and the carbons C-5 (*δ* 143.49 (C) and C-6 (*δ* 125.49 (CH)) of the double bond and the vicinal coupling of H-7 with H-6 (*δ* 5.27) in the HHCOSY. The NOESY spectrum revealed correlations between H-7 and the axial H-9 (*δ* 1.07) and H-14 (*δ* 1.27) indicating that all these protons are in *axial* position and logically consistent the hydroxyl group at C-7 is equatorial and therefore in *β*-orientation. Therefore its NMR data were in good accordance to those of the close related *3β, 7β-dihydroxyandrost-5-en-17-one.*

#### 3β, 7β-dihydroxypregn-5-en-20-one (7β-OH-PREG)

^1^H NMR: (CDCl_3_, 500 MHz): *δ* 0.63 s (3H, 3 x H-18), 1.03 *s* (3H, 3 x H-19), 1.06 *m* (H-1a), 1.07 *m* (H-9), 1.27 *m* (H-14), 1.39 *m* (H-12b), 1.40 *m* (H-8), 1.47 *m* (H-11a), 1.50 *m* (H-2a), 1.56 *m* (H-15a), 1.62 *m* (H-11b), 1.70 *m* (H-16a), 1.84 *m* (2H, H-1b and H-2b), 1.92 *m* (H-15b), 2.03 *m* (H-12b), 2.11 *s* (3H, 3x H-21), 2.18 *m* (H-16b), 2.23 *m* (H-4a), 2.32 *ddd* (*J* = 13.5, 5.0 and 2.0 Hz, H-4b), 2.48 *dd* (*J* = 9.5 and 9.5 Hz, H-17), 3.53 *m* (H-3), 3.83 *ddd* (*J* = 8.0, 2.0 and 2.0 Hz, H-7), 5.27 *dd* (*J* = 2.0 and 2.0 Hz, H-6). ^13^C NMR (CDCl_3_, 125 MHz): δ 13.20 (C-18), 19.14 (C-19), 21.04 (C-11), 23.35 (C-16), 26.68 (C-15), 31.53 (C-2), 31.59 (C-21), 36.58 (C-10), 36.91 (C-1), 38.64 (C-12), 41.09 (C-8), 41.49 (C-4), 44.42 (C-13), 48.12 (C-9), 56.03 (C-14), 63.05 (C-17), 71.36 (C-3), 73.14 (C-7), 125.49 (C-6), 143.49 (C-5), 209.54 (C-20).

## Abbreviations

P450: Cytochrome P450 enzymes; AdR: Adrenodoxin reductase; Adx: Adrenodoxin; *B. megaterium*: *Bacillus megaterium*; DHEA: Dehydroepiandrosterone; PREG: Pregnenolone; HEPES: (4-(2-hydroxyethyl)-1-piperazineethanesulfonic acid); NMR: Nuclear spin resonance; ppm: Parts per million; Rf: Retention factor; HPLC: High-performance liquid chromatography; TLC: Thin layer chromatography.

## Competing interests

The authors declare that there are no competing interests.

## Authors’ contributions

DS carried out the biochemical and biotechnological experiments and drafted the manuscript. JZ did all NMR measurements and structure determinations of the hydroxysteroids produced in this work. RB participated in the interpretation of the results and assisted in manuscript drafting. All authors read and approved the final manuscript.

## Supplementary Material

Additional file 1: Figure S1 Dexamethasone conversion with *B. megaterium* ATCC13368: Dexamethasone (200 μM) was added to 500 μl sample of a 16 h culture of *B. megaterium* ATCC13368 wild type and cyp106a2-knockout and incubated for 60 minutes at 30°C. **Figure S2.** Prednisone conversion with *B. megaterium* ATCC13368: Prednisone (200 μM) was added to 500 μl sample of a 16 h culture of B. megaterium ATCC13368 wild type and cyp106a2-knockout and incubated for 60 minutes at 30°C. **Figure S3.** Digitoxigenin conversion with *B. megaterium* ATCC13368: Digitoxigenin (200 μM) was added to 500 μl sample of a 16 h culture of B. megaterium ATCC13368 wild type and cyp106a2-knockout and incubated for 60 minutes at 30°C. **Figure S4.** Conversion of DHEA with the *B. megaterium* MS941 strain without expression plasmid: Conversions were conducted in 50 mM potassium-phosphate buffer adjusted to pH 7.4, at 30°C and 150 rpm in 300 ml baffled shake flasks. 400 μM DHEA were added and samples taken at indicated time-points. No remarkable DHEA conversion was detected after 24 hours. The impurity eluting at about minute 1, is most probably a metabolite from the *B. megaterium* MS941 cells, independent from CYP106A2 dependent DHEA conversion. **Figure S5.** Selectivity of DHEA-hydroxylation with the *B. megaterium* ATCC13368 wild type strain compared with the *B. megaterium* MS941_pSMF2.1CAA overexpression strain: Conversions were performed with 24 h cultures of both strains for 60 minutes in 50 mM potassium-phosphate buffer and DHEA. The amount of the respective products in percent was calculated using the peak area/min. The bars show the mean values and standard deviations from three independent conversions. Click here for file
