# Supplementary material for: Steroid conversion with CYP106A2 – production of pharmaceutically interesting DHEA metabolites
Source: Microb Cell Fact. 2014 Jun 5;13:81. doi: 10.1186/1475-2859-13-81 (PMC4080778; doi:10.1186/1475-2859-13-81)
Supplement: Additional file 1: Figure S1 — Dexamethasone conversion with B. megaterium ATCC13368: Dexamethasone (200 μM) was added to 500 μl sample of a 16 h culture of B. megaterium ATCC13368 wild type and cyp106a2-knockout and incubated for 60 minutes at 30°C. Figure S2. Prednisone conversion with B. megaterium ATCC13368: Prednisone (200 μM) was added to 500 μl sample of a 16 h culture of B. megaterium ATCC13368 wild type and cyp106a2-knockout and incubated for 60 minutes at 30°C. Figure S3. Digitoxigenin conversion with B. megaterium ATCC13368: Digitoxigenin (200 μM) was added to 500 μl sample of a 16 h culture of B. megaterium ATCC13368 wild type and cyp106a2-knockout and incubated for 60 minutes at 30°C. Figure S4. Conversion of DHEA with the B. megaterium MS941 strain without expression plasmid: Conversions were conducted in 50 mM potassium-phosphate buffer adjusted to pH 7.4, at 30°C and 150 rpm in 300 ml baffled shake flasks. 400 μM DHEA were added and samples taken at indicated time-points. No remarkable DHEA conversion was detected after 24 hours. The impurity eluting at about minute 1, is most probably a metabolite from the B. megaterium MS941 cells, independent from CYP106A2 dependent DHEA conversion. Figure S5. Selectivity of DHEA-hydroxylation with the B. megaterium ATCC13368 wild type strain compared with the B. megaterium MS941_pSMF2.1CAA overexpression strain: Conversions were performed with 24 h cultures of both strains for 60 minutes in 50 mM potassium-phosphate buffer and DHEA. The amount of the respective products in percent was calculated using the peak area/min. The bars show the mean values and standard deviations from three independent conversions. [file 1475-2859-13-81-S1.pdf]

Additional material:

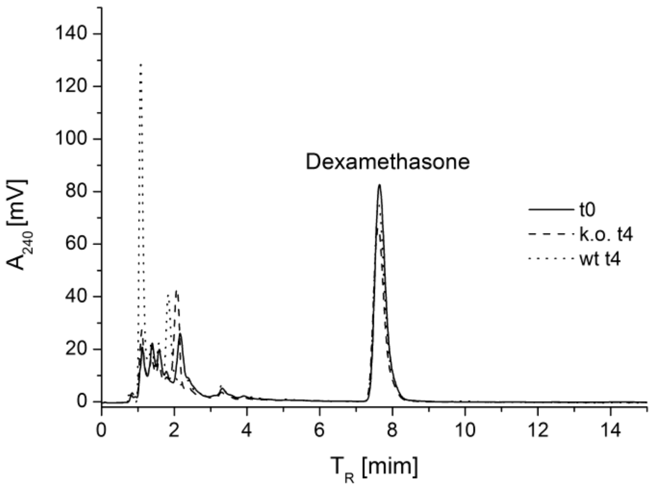

Figure S1: HPLC chromatogram of dexamethasone conversion with *B. megaterium* ATCC13368

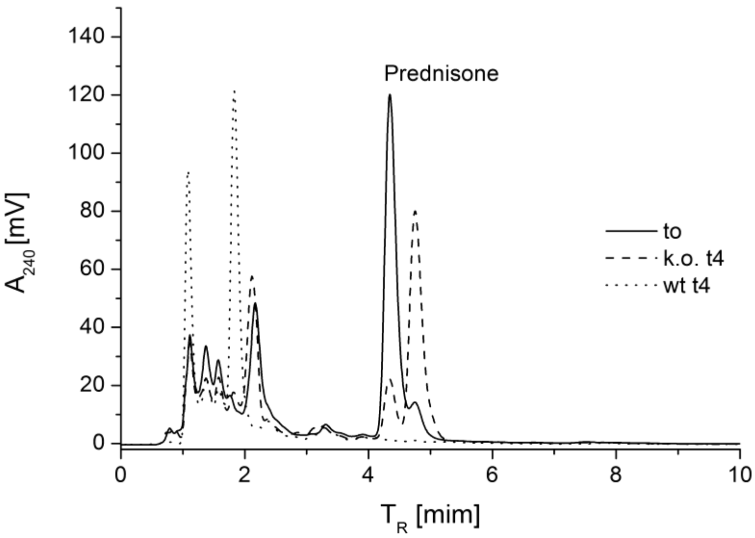

Figure S2: HPLC-chromatogram of prednisone conversion with B. megaterium ATCC13368

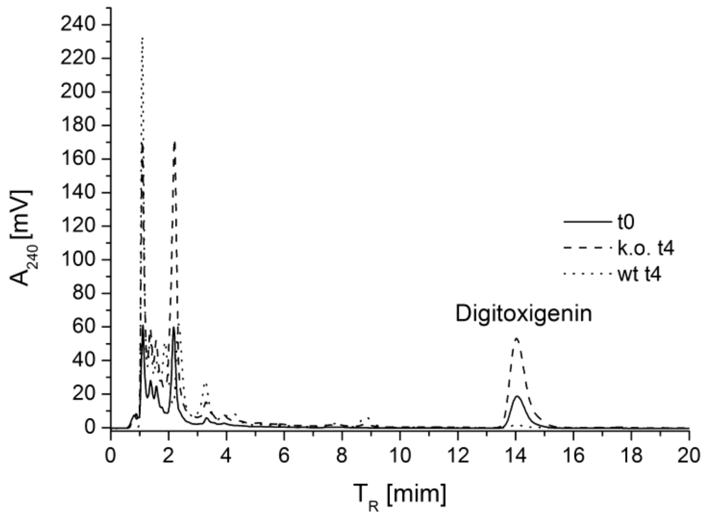

Figure S3: HPLC-chromatogram of digitoxigenin conversion with *B. megaterium* ATCC13368

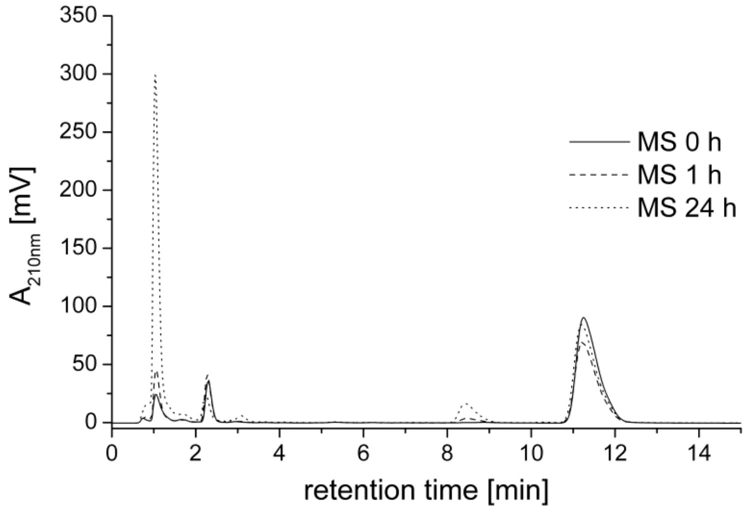

Figure S4: HPLC-chromatogram of DHEA-conversion with *B. megaterium* MS941 without expression plasmid.

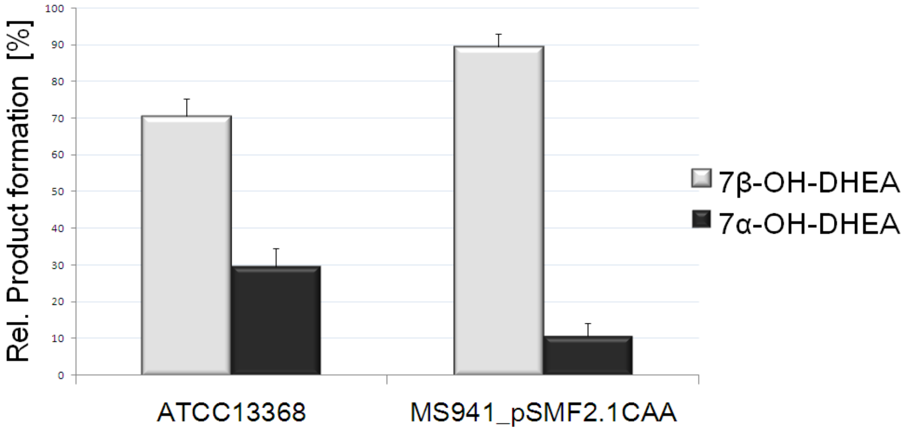

Figure S5: Selectivity of DHEA-hydroxylation with the *B. megaterium* ATCC13368 wild type strain compared with the *B. megaterium* MS941_pSMF2.1CAA overexpression strain
